# Conventional versus modified application of COOK Cervical Ripening Balloon for induction of labor at term: a randomized controlled trial

**DOI:** 10.1186/s12884-022-05035-w

**Published:** 2022-10-02

**Authors:** Chaoyue Wen, Xuemin Liu, Ying Wang, Jun Wang

**Affiliations:** Department of Obstetrics and Gynecology, Shengjing Hospital of China Medical University, Shenyang, Liaoning, China

**Keywords:** Cervical ripening, Induction of labor, COOK Cervical Ripening Balloon, Term pregnancy

## Abstract

**Background:**

This study aims to evaluate the efficacy and safety of the modified application of COOK Cervical Ripening Balloon (CCRB) for induction of labor (IOL) at term in primipara.

**Methods:**

A total of 227 singleton full-term pregnancies with indications of IOL were enrolled and randomly divided into the control and study groups in our hospital from January 2021 to December 2021. In the control group, a conventional method was used. Both the uterine and vaginal balloons were filled to 80 mL and removed after 12 h. In the study group, a modified method was used. The uterine and vaginal balloons were filled to 120 mL and 40 mL respectively. Light traction was given to help CCRB to be discharged after 12 h placement. Oxytocin was administered in both groups after CCRB was discharged before labor starting. The improved Bishop scores, duration of labor, and spontaneous delivery rate were evaluated in the two groups.

**Results:**

The improved Bishop scores in the study group were 3.06 ± 0.97 at 12 h placement of CCRB and 4.37 ± 0.87 when CCRB was discharged, which were significantly higher compared to the control group (2.52 ± 0.79, *p* < 0.05). Duration of the first stage of labor and the full labor in the study group were significantly shorter than those in the control group ((6.17 ± 2.85) h vs. (7.27 ± 2.90) h, *p* = 0.010; (7.07 ± 3.18) h vs. (8.09 ± 3.11) h, *p* = 0.028). No difference in spontaneous delivery rate between the two groups was observed. But the delivery rate within 24 h between the two groups was significantly different (79.79% vs. 55.91%, *p* < 0.05). For the cases with initial Bishop scores ≤ 3, the improved score was significantly increased, the first stage of labor and the full labor were significantly shorter in the study group than those in the control group (*p* < 0.05). Those results were not observed in cases with initial Bishop scores of 4–6.

**Conclusions:**

The modified application of CCRB could benefit cervical ripening, shorten the duration of labor, especially for cases with poor cervical maturity, and improve the delivery rate within 24 h.

**Trial registration:**

Retrospectively registered: ChiCTR2200058270. Registered 04/04/2022.

## Background

Induction of labor (IOL) is the artificial initiation of labor before its spontaneous onset to achieve vaginal delivery [1]. IOL at term is mainly applied to pregnant women with some comorbidities that require termination of pregnancy. In recent years, some research has shown that IOL after 39 gestational weeks without medical indication can lower the risk of cesarean section [2, 3]. In the United States, the rate of IOL in primipara increased from 22.5% in 2006 to 42.9% in 2012 [4, 5]. The rate of IOL at term in China also increased from 20.4% to more than 30% in 2013 [6]. Therefore, it is particularly important to choose an effective and safe method of IOL.

Cervical maturity evaluated with the Bishop score is the key to predicting the outcome of IOL. Induction to active labor is usually successful with a score of 9 or greater and often fails with a score less than 6 [5]. At present, commonly used cervical ripening methods include pharmacological methods represented by prostaglandin E2 and mechanical methods represented by COOK Cervical Ripening Balloon (CCRB). According to literature reports, there is no significant difference in the spontaneous delivery rate between the two methods [7–9]. Compared with pharmacological methods, mechanical methods are preferred for cervical ripening with fewer pain scores and less uterine hyperstimulation [8, 10–12]. The mechanical methods induce uterine contractions mainly by squeezing the internal cervical os through a uterine balloon, which stretches the lower uterine segment, thereby promoting the local release of prostaglandins [13]. Besides the local effect, the balloon can also promote uterine contractions through neuro-endocrine reflexes (such as the Ferguson reflex) [14].

CCRB is currently the most used cervical ripening double-balloon device clinically. Besides the uterine balloon, CCRB has another balloon named vaginal balloon, which helps to fix the uterine balloon on the lower uterine segment. However, the success rate of spontaneous delivery after cervical ripening with CCRB is about 66%-70% according to the manufacturer’s instructions [7, 8]. In this study, we evaluate a modified operating procedure of CCRB in the aim of achieving a better cervical ripening effect and improving the success rate of spontaneous delivery.

## Methods

### Selection and grouping of patients

In this study, 227 pregnancies were selected from 976 hospitalized singleton full-term patients ready for IOL and were randomly divided into the control group and the study group in the Shengjing Affiliated Hospital of China Medical University from January 2021 to December 2021.

Inclusion criteria included: (1) primiparous pregnancy; (2) cephalic presentation; (3) NST reaction type; (4) cervical Bishop score ≤ 6.

**Fig. 1:**
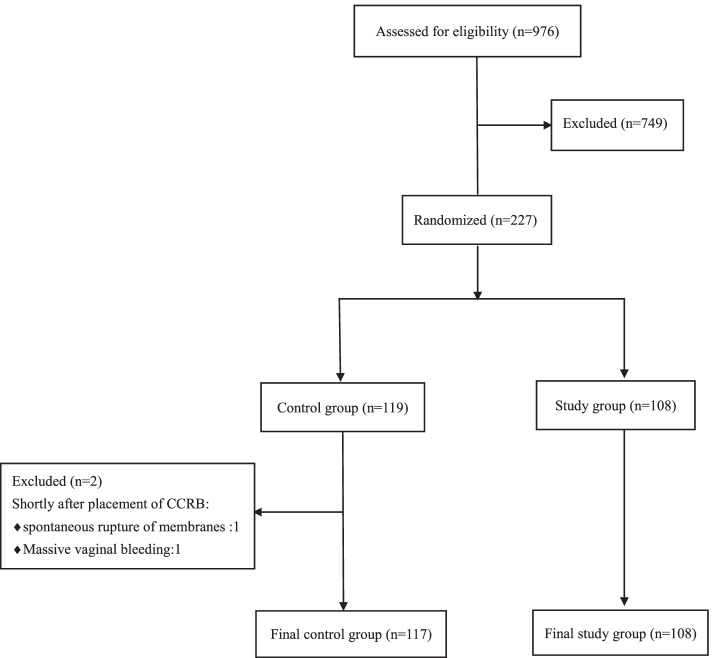
Flowchart demonstrating

Exclusion criteria included (1) placenta previa; (2) rupture of membranes; (3) hydramnios; (4) scarred uterus; (5) fetal malformation or intrauterine fetal death; (6) combined vaginitis; (7) history of late abortion or premature delivery; (8) presence of contraindications to vaginal delivery (Fig. 1).

### Ethical approval and patient consent.

The study protocol was approved by the Medical Ethics Committee of the Shengjing Affiliated Hospital of China Medical University (Ethics No. 2021PS248J) and all patients signed informed consent forms.

### Study methods and operating procedures

**Fig. 2:**
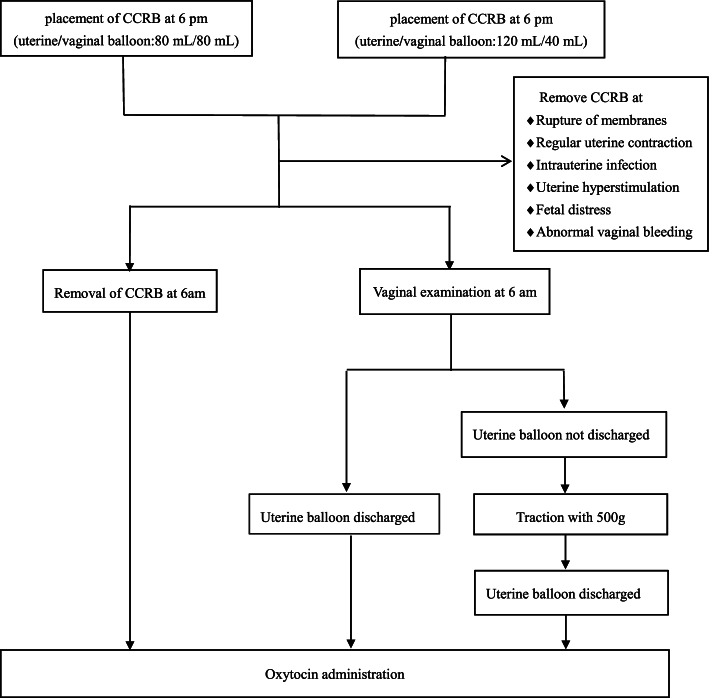
Operating procedures for the control group (left) and the study group (right)

CCRB (Type J-CRBS-184000, Spencer, IN, USA) was uniformly used in the present study. The detailed operating procedures of the two groups were as follows (Fig. 2).

On the first day, CCRB was placed at 6 pm. CCRB catheter was placed through the cervical canal to the internal cervical os after strict sterilization without touching the vaginal wall. The uterine balloon was filled with 40 mL saline and pulled back until the uterine balloon was against the internal cervical os and the vaginal balloon was set outside the external cervical os. The vaginal balloon was filled with 20 mL saline so that the two balloons were respectively located on the inner and outer sides of the cervix. Then saline was further injected until the uterine and vaginal balloon reached the target volume.

The target volumes of the control group are 80 mL in the uterine balloon and 80 mL in the vaginal balloon.

The target volumes of the study group are 120 mL in the uterine balloon and 40 mL in the vaginal balloon.

On the second day, the Bishop score was evaluated and an electronic fetal monitor was given at 6 am.

For the control group, CCRB was removed if the uterine balloon was not discharged during the night.

For the study group, 500 g weight traction of the catheter was given if the uterine balloon was not discharged at 6 am. The Bishop score was evaluated again when the uterine balloon was discharged.

In both groups, fetal heart rate and uterine contractions were observed for an hour after removal of CCRB. If labor had not started, oxytocin was administered to promote contractions. And the timing of artificial rupture of membranes was decided by the assessment of obstetricians.

The oxytocin administration was performed as the conventional oxytocin labor induction. Briefly, a concentration of 0.5% oxytocin was given intravenously 8 drops per minute (8 drops/min), if type I electronic fetal monitoring with no regular uterine contractions was traced. The fetal heart rate was monitored and the titer of oxytocin was increased by 8 drops/min every 30 min until regular uterine contractions occurred. The maximum titer of oxytocin was 40 drops/min, and the maximum concentration was 1%. If labor was not initiated at 5 pm, oxytocin administration was stopped and performed again at 6 am on the following days until delivery.

CCRB was removed if the following conditions occurred: (1) spontaneous rupture of membranes; (2) regular uterine contractions; (3) signs of intrauterine infection; (4) uterine hyperstimulation; (5) fetal distress; (6) abnormal bleeding suspicious for placental abruption.

### Observation indicators

The following characteristics were recorded: age, gravidity, height, body weight, body mass index (BMI, calculated as weight in kilograms divided by height in meters squared), gestational age, indications of IOL, initial Bishop scores, neonatal birth weight, and time of rupture of membranes.

The following primary outcomes were recorded: the Bishop scores at 6 am in both groups, the Bishop score when the uterine balloon was discharged in the study group, modes of delivery, indications of cesarean section, duration of first stage and full labor, the induction-delivery time (time from placement of CCRB to vaginal delivery), successful induction rate = number of cases entering active phase within 72 h (cases where women entered active phase but for whom birth was by cesarean section due to abnormal stage of labor or fetal heart rate, were nevertheless considered effective) /total number, spontaneous delivery rate = vaginal delivery number/ total number (including cesarean delivery), delivery rate within 24 h = the induction-delivery time within 24 h number/ total number (excluding cesarean delivery).

The following secondary outcomes were recorded: 1-min and 5-min neonatal Apgar scores, postpartum hemorrhage, cervical laceration, fetal distress, and other delivery complications, midwifery and modes of midwifery, labor analgesia, and pain scores from placement of CCRB to removal (using the visual analog scale, VAS:0–10, 0 = no pain, 10 = worst pain possible).

### Statistical methods

To determine the proper sample size, statistical power analysis was conducted using GPower 3.1 with an α risk of 0.05 and a β risk of 0.05. The final calculated sample size was 106.

SPSS 26.0 statistical software was used to analyze the differences between the two groups.

The values and variables are reported as the means ± standard deviation. The student’s T-test was performed to compare the variables in a Gaussian distribution. The chi-square test was used to evaluate the categorical variables. The Wilcoxon test was used to evaluate the difference in a non-Gaussian distribution between the two groups. Kaplan–Meier survival curve was used to analyze the cumulative number of successful vaginal deliveries in the two groups at different times. Log-Rank test was used to compare the difference between the two groups. "Vaginal delivery" was used as the observation endpoint. *P* < 0.05 was considered statistically significant.

## Results

A total of 227 pregnant women who met the criteria participated in this study, with 119 initially assigned in the control group and 108 in the study group. In the control group, 2 cases were excluded later; one case had spontaneous rupture of membranes, the other case had heavy vaginal bleeding shortly after placement of CCRB (this case was later diagnosed with placenta previa after emergent cesarean section). Therefore, data from the 117 cases in the control group and 108 cases in the study group were further analyzed and reported (Fig. 1).

### Baseline clinical characteristics

The age, gravidity, height, body weight, BMI, gestational age, indications of IOL, initial Bishop scores, and neonatal birth weight were compared. There were no significant differences in basic clinical characteristics**,** indications of IOL, and neonatal birth weight between the two groups (Table 1).

**Table 1:** Baseline clinical characteristics of the two groups

Variable	Control group (*n* = 117)	Study group (*n* = 108)	*p* value
Age (y,‾X ± s)	30.54 ± 3.15	30.21 ± 3.71	0.478
Gravidity (n, min–max)	1–3	1–3	0.265
Height (m,‾X ± s)	1.64 ± 0.59	1.65 ± 0.48	0.777
Body weight (kg,‾X ± s)	75.29 ± 10.05	73.45 ± 9.86	0.168
BMI (kg/m^2^,‾X ± s)	27.85 ± 3.50	27.11 ± 3.64	0.123
Gestational age (week,‾X ± s)	39.85 ± 0.85	39.93 ± 0.71	0.425
Indications of IOL, n(%)			
Delayed pregnancy	47 (40.17)	41 (37.96)	0.735
Oligohydramnios	35 (29.91)	33 (30.56)	0.917
HDP	23 (19.66)	18 (16.67)	0.561
PGDM and GDM	8 (6.84)	10 (9.26)	0.504
Suspected macrosomia	4 (3.42)	6 (5.56)	0.437
Initial Bishop scores (‾X ± s)	2.85 ± 0.97	2.99 ± 1.09	0.294
Birth weight (g,‾X ± s)	3392.09 ± 356.46	3408.06 ± 378.66	0.745

*Abbreviations*: *BMI* Body mass index, *HDP* Hypertensive disorders of pregnancy, *PGDM* Pregestational diabetes mellitus, *GDM* Gestational diabetes mellitus

Student’s T-test, Chi-square test, and Wilcoxon test were used

### Primary outcomes

We observed that the uterine balloons were discharged in 25.6% cases of the control group and 20.4% cases of the study group at 6 am of the next day. The improvement of the Bishop score, successful induction rate, spontaneous delivery rate, cesarean section rate, and the indications were compared between the two groups (Table 2).

**Table 2:** Primary outcomes of the two groups

Variable	Control group (*n* = 117)	Study group (*n* = 108)	*p* value
CCRB was discharged within 12 h, n (%)	30 (25.6)	22 (20.4)	0.349
The improved Bishop scores at 12 h (‾X ± s)			
Total scores	2.52 ± 0.79	3.06 ± 0.97	0.000*
Dilation	1.17 ± 0.38	1.62 ± 0.51	0.000*
Cervical consistency	0.91 ± 0.32	0.94 ± 0.27	0.332
Effacement	0.60 ± 0.49	0.66 ± 0.48	0.362
Position of cervix	0.18 ± 0.39	0.19 ± 0.40	0.775
Station	-0.33 ± 0.50	-0.36 ± 0.57	0.696
The improved Bishop scores when CCRB was discharged (‾X ± s)			
Total scores	2.52 ± 0.79	4.37 ± 0.87	0.000*
Dilation	1.17 ± 0.38	2.03 ± 0.21	0.000*
Cervical consistency	0.91 ± 0.32	1.00 ± 0.36	0.040*
Effacement	0.60 ± 0.49	1.30 ± 0.65	0.000*
Position of cervix	0.18 ± 0.39	0.25 ± 0.44	0.201
Station	-0.33 ± 0.50	-0.20 ± 0.47	0.044
Successful induction rate, n (%)	112 (95.73)	108(100)	0.030*
Spontaneous delivery rate, n (%)	93 (79.49)	94(87.04)	0.131
Cesarean section rate, n (%)	24 (20.51)	14(12.96)	0.131
Indications of cesarean section, n (%)			
Abnormal fetal heart rate	10 (8.55)	9(8.33)	0.954
Abnormal stage of labor	6 (5.13)	4 (3.70)	0.604
Psychological factors	8 (6.84)	1 (0.93)	0.024*

Student’s T-test, Chi-square test, and Wilcoxon test were used

^*^ *p* < 0.05 was considered statistically significant

The improved Bishop scores at 12 h after the placement of CCRB in the study group was significantly higher than that of the control group (3.06 ± 0.97 vs. 2.52 ± 0.79, *p* = 0.000), and the improvement was even larger when CCRB was discharged (4.37 ± 0.87 vs. 2.52 ± 0.79, *p* = 0.000).

By analyzing the five scoring items of the Bishop score respectively, we found that in both groups, the dilation item was increased by more than 1 point, the most in the five items, followed by the cervical consistency item, while the effacement and position of cervix items increased less, and the station item score even decreased.

Similar improvements were observed in the study group when CCRB was discharged, except the total score and dilation item score improved more than those at 12 h and the improvement of the effacement score came to second place in the study group. The successful induction rate in the study group was significantly higher than that in the control group (100% vs 95.73%, *p* = 0.030). The spontaneous delivery rate in the study group was higher than that in the control group (87.04% vs 79.49%), but there was no significant difference (*p* = 0.131). The indications of cesarean section in two groups were also analyzed. With 14 cases undergoing cesarean section in the study group, 9 for fetal distress, 4 for the abnormal stage of labor (2 for arrested active phase, 1 for arrested descent, and 1 for protracted second stage), and 1 for maternal loss of confidence due to long induction time. In the control group, 24 cases underwent cesarean section, with 10 for fetal distress, 6 for abnormal stage of labor (3 for arrested active phase, 2 for arrested descent, and 1 for protracted second stage), and 8 for losing confidence due to long induction time. The cases of cesarean section due to psychological factors in the control group were significantly higher than that in the study group (6.84% vs. 0.93%, *p* = 0.024).

### Delivery process

Eventually, 93 cases in the control group and 94 cases in the study group were spontaneous delivery. The process of delivery was compared between the two groups (Table 3).

**Table 3:** Delivery process of the two groups

Variable	Control group (*n* = 93)	Study group (*n* = 94)	*p* value
First stage of labor (h,‾X ± s)	7.27 ± 2.90	6.17 ± 2.85	0.010*
Total stage of labor (h, ‾X ± s)	8.09 ± 3.11	7.07 ± 3.18	0.028*
Delivery rate within 24 h, n (%)	52 (55.91)	75 (79.79)	0.000*
Pain scores during placement of CCRB (‾X ± s)	3.94 ± 1.15	4.10 ± 1.33	0.379
Labor analgesia during labor, n (%)	33 (35.48)	21 (22.34)	0.047*

Student’s T-test and Chi-square test were used

^*^ *p* < 0.05 was considered statistically significant

The durations of first stage of labor and full labor in the study group were significantly shorter than those of the control group (for the first stage (6.17 ± 2.85) h vs. (7.27 ± 2.90) h, *p* = 0.010; for the full labor (7.07 ± 3.18) h vs. (8.09 ± 3.11) h, *p* = 0.028).

Delivery rate within 24 h was significantly higher in the study group than that in the control group (79.79% vs. 55.91%, *p* < 0.05).

There was no significant difference in the pain scores during placement of CCRB between the two groups (4.10 ± 1.33 vs. 3.94 ± 1.15, *p* = 0.379). However, the use of labor analgesia in the control group was significantly higher than that in the study group (35.48% vs. 22.34%; *p* = 0.047).

**Fig. 3:**
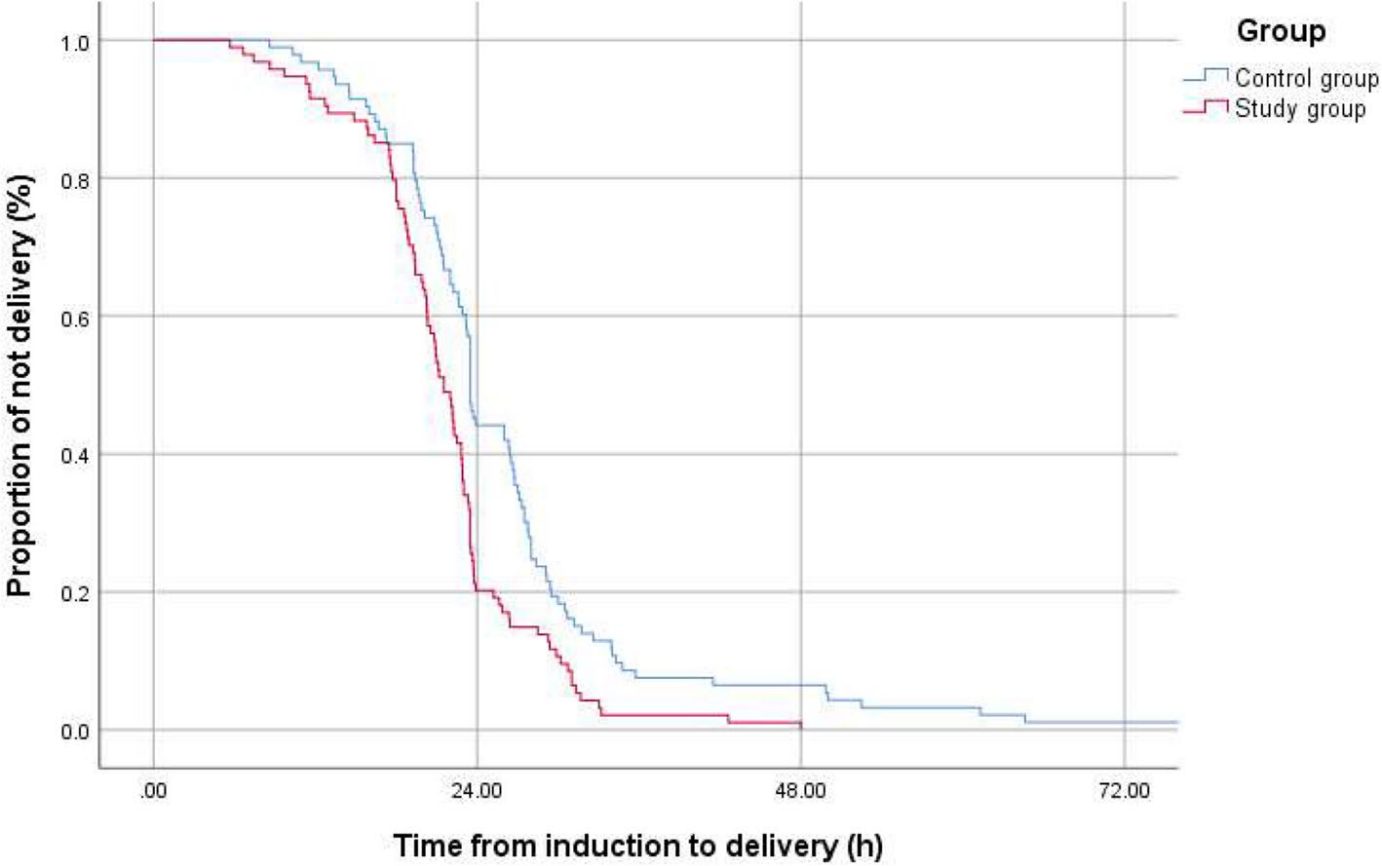
Remaining not delivery cases at the time from placement of CCRB to vaginal delivery (excluding cesarean delivery cases)

Kaplan–Meier curves were used to compare cumulative successful vaginal delivery rates between the two groups. All cases achieved vaginal delivery within 48 h in the study group while there were still a few cases not achieving successful vaginal delivery after 72 h in the control group. The median time from placement of CCRB to vaginal delivery in the study group was significantly shorter than that of the control group (Log-Rank test, *p* < 0.001) (Fig. 3).

### Cervical ripening effects with different initial Bishop scores

To find the ripening effects of the modified method on the cervix with different initial maturities, we stratified the cases of the two groups with initial Bishop scores (Table 4).

**Table 4:** Cervical ripening effects with different initial Bishop scores

Variable	Initial Bishop scores ≤ 3			Initial Bishop scores 4–6		
	Control group (*n* = 66)	Study group (*n* = 64)	*p* value	Control group (*n* = 27)	Study group (*n* = 30)	*p* value
The improved Bishop scores at 12 h (‾X ± s)						
Total scores	2.56 ± 0.70	3.11 ± 0.99	0.000*	2.41 ± 0.89	2.93 ± 1.08	0.051
Dilation	1.11 ± 0.31	1.53 ± 0.50	0.000*	1.33 ± 0.48	1.83 ± 0.46	0.000*
Cervical consistency	0.95 ± 0.27	1.02 ± 0.13	0.106	0.78 ± 0.42	0.80 ± 0.41	0.841
Effacement	0.61 ± 0.49	0.61 ± 0.49	0.969	0.59 ± 0.50	0.73 ± 0.45	0.271
Position of cervix	0.20 ± 0.40	0.20 ± 0.41	0.931	0.11 ± 0.32	0.20 ± 0.41	0.367
Station	-0.30 ± 0.46	-0.25 ± 0.44	0.503	-0.41 ± 0.57	-0.63 ± 0.72	0.198
The improved Bishop scores when CCRB was discharged (‾X ± s)						
Total scores	2.56 ± 0.70	4.56 ± 0.87	0.000*	2.41 ± 0.89	3.93 ± 0.74	0.000*
Dilation	1.11 ± 0.31	2.03 ± 0.25	0.000*	1.33 ± 0.48	2.03 ± 0.18	0.000*
Cervical consistency	0.95 ± 0.27	1.09 ± 0.29	0.006*	0.78 ± 0.42	0.80 ± 0.41	0.841
Effacement	0.61 ± 0.49	1.33 ± 0.69	0.000*	0.59 ± 0.50	1.23 ± 0.57	0.000*
Position of cervix	0.20 ± 0.40	0.28 ± 0.45	0.264	0.11 ± 0.32	0.23 ± 0.43	0.226
Station	-0.30 ± 0.46	-0.17 ± 0.42	0.093	-0.41 ± 0.57	-0.37 ± 0.56	0.786
First stage of labor (h, ‾X ± s)	7.72 ± 2.77	6.42 ± 2.86	0.009*	6.16 ± 2.95	5.64 ± 2.82	0.502
Total stage of labor (h, ‾X ± s)	8.52 ± 3.09	7.36 ± 3.20	0.036*	7.02 ± 2.93	6.47 ± 3.11	0.494

Student’s T-test was used

^*^ *p* < 0.05 was considered statistically significant

There were 187 cases achieving vaginal delivery in this study. For the 130 cases with initial Bishop scores ≤ 3 points, 66 cases of the control group and 64 cases of the study group achieved vaginal delivery. For the remaining 57 cases with initial Bishop scores of 4–6 points, 27 cases in the control group and 30 cases in the study group achieved vaginal delivery.

For patients with the initial Bishop scores ≤ 3 points, the Bishop score improved significantly in the study group compared with that in the control group both at 12 h of placement and when CCRB was discharged (*p* = 0.000, *p* = 0.000). The durations of the first stage and full labor were significantly shorter than those in the control group (*p* = 0.009, *p* = 0.036).

For those with the initial Bishop scores of 4–6 points, the improvement of the Bishop score in the study group was significantly higher than that in the control group when CCRB was discharged (*p* = 0.000). There was no significant difference in the improvement of the Bishop score at 12 h of placement, the first stage of labor, or the total stage of labor in the study group compared with the control group (*p* > 0.05).

Similarly, each assigned item of the Bishop score was analyzed. The dilatation score improved the most, the cervical consistency score improved the second, and the effacement and position score of the cervix improved the least, while the fetal presentation position score even decreased in both groups whether the initial Bishop scores were high or low at 12 h of placement. When CCRB was discharged, the improvement of the effacement score came to second place in the study group.

### Delivery-related risks and outcomes

The occurrence of delivery-related risks and outcomes in the two groups were analyzed (Table 5). There were no significant differences in the incidence of placental abruption, intrauterine infection, postpartum hemorrhage, cervical laceration, perineal laceration, forceps delivery, episiotomy, umbilical cord prolapse, neonatal hypoxic or asphyxia, and neonatal death between the two groups (*p* > 0.05).

**Table 5:** Delivery-related risks and outcomes of the two groups

Variable	Control group (*n* = 93)	Study group (*n* = 94)	*p* value
Placental abruption, n (%)	0	0	-
Intrauterine infection, n (%)	2(2.15)	2(2.13)	0.991
Postpartum hemorrhage, n (%)	7(7.53)	5(5.32)	0.538
Cervical laceration, n (%)	1(1.08)	2(2.13)	0.567
Perineal laceration, n (%)	10(11.11)	19(20.21)	0.074
Forceps delivery, n (%)	3(3.23)	4(4.26)	0.711
Episiotomy, n (%)	79(84.95)	69(73.40)	0.052
Umbilical cord prolapse, n (%)	0	0	-
Apgar score (min–max)			
1-min score	9 -10	9 -10	0.286
5-min score	10–10	10–10	-
Neonatal hypoxic or asphyxia, n (%)	0	0	-
Neonatal death, n (%)	0	0	-

Chi-square test and Wilcoxon test were used

### Effects of different timing of rupture of membranes on the delivery process

We then analyzed the effects of different timing of rupture of membranes on the delivery process (Table 6).

**Table 6:** Effects of the different timing of rupture of membranes on the delivery process

Group	n	First stage (h)	Total stage (h)	Induction-delivery time (h)
Control group	93			
Before regular contractions^#^	22	6.23 ± 1.60	7.04 ± 1.68	25.20 ± 13.03
After regular contractions^#^	71	7.59 ± 3.13	8.41 ± 3.37	29.75 ± 9.83
* p* value		0.009*	0.070	0.135
Study group	94			
Before regular contractions^#^	26	6.12 ± 2.77	6.90 ± 2.97	20.38 ± 5.32
After regular contractions^#^	68	6.28 ± 3.11	7.53 ± 3.70	25.00 ± 8.60
* p* value		0.816	0.390	0.015*

Student’s T-test was used

^*^ *p* < 0.05 was considered statistically significant

“^#^” amniotomy or spontaneous rupture of membranes before or after regular contractions

In the control group, the first stage of labor was (6.23 ± 1.60) h if amniotomy or spontaneous rupture of membranes occurred before regular contractions, and was (7.59 ± 3.13) h if membranes raptured after regular contractions; the difference in the lengths of first stage was significant (*p* = 0.009). There were no significant differences in either the length of full labor or the induction-delivery time, regardless of the timing of membranes rupture (before or after regular contractions).

In the study group, the induction-delivery time with membranes ruptured before regular contractions was significantly shorter than that with membranes ruptured after regular contractions ((20.38 ± 5.32) h vs. (25.00 ± 8.60) h, *p* = 0.015). There were no significant differences in the length of either first stage or full labor, regardless of the timing of membranes rupture (before or after regular contractions).

## Discussion

IOL in late pregnancy can reduce the occurrence of post-term pregnancy and post-term related complications, and protect the fetus from adverse pregnancy outcomes [8, 15]. The World Health Organization recommends delivery rate within 24 h as a key indicator for evaluating the effects of IOL [16]. At present, cervical ripening with CCRB combined with oxytocin administration is a common clinical method of IOL. The delivery rate within 24 h of the recommended procedure is about 69% [17]. For those with poor cervical maturity, the cervical ripening effect is even more unsatisfactory. In our study, according to the CCRB manufacturer’s instructions, the delivery rate within 24 h was 55.91% in the control group, slightly lower than that reported by previous research, which might be related to the relatively lower initial Bishop scores of this group of patients. However, the delivery rate reached 79.79% within 24 h and 100% within 48 h in the study group with our modified procedure, which were significantly higher than those of the control group. These results suggest the modified procedure was more efficient than the manufacturer-recommended protocol.

**Fig. 4:**
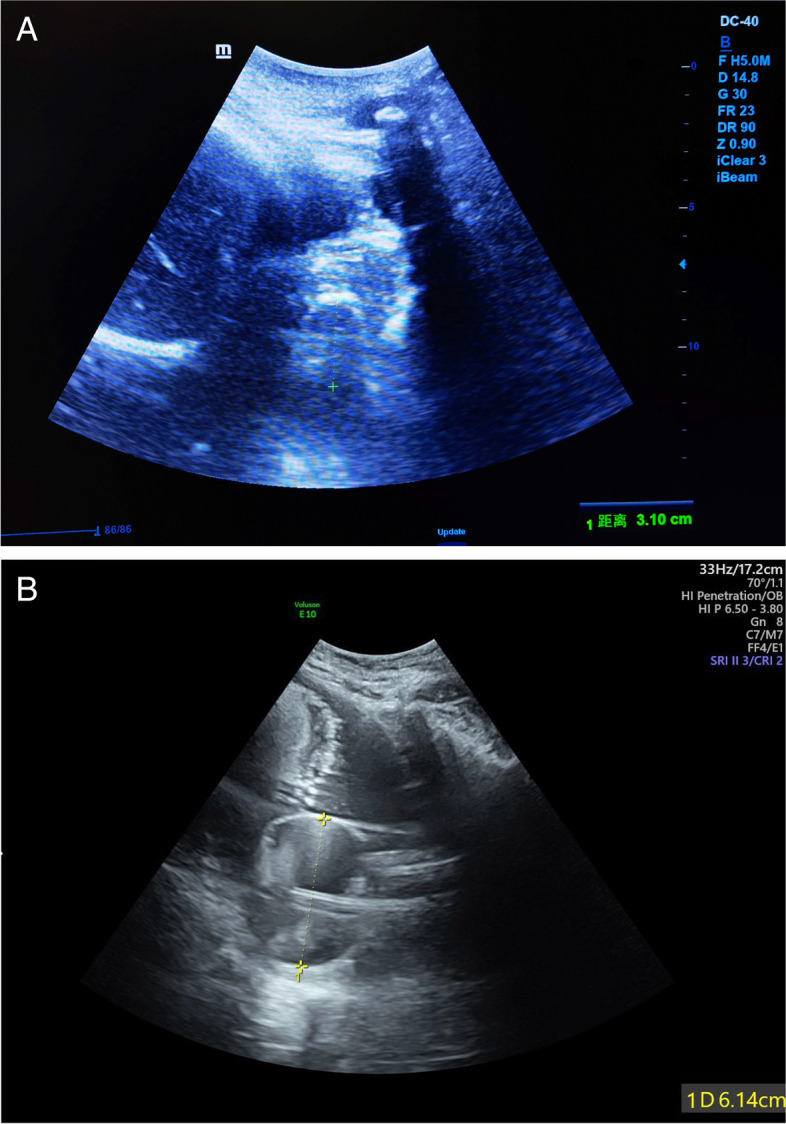
Ultrasonic images of the uterine balloon with different volumes. **A**: Control group; **B**: Study group

A related study pointed out that the Bishop score after cervical ripening can better predict the duration of labor than initial Bishop scores [18]. We observed that CCRB improved the Bishop score mainly in the dilation item and cervical consistency item in our study. For the station item, the score even decreased due to fetal head being pushed up by the uterine balloon, especially in patients with low initial Bishop scores. The uterine balloon of CCRB was about 3 cm in diameter when filled to 80 mL (Fig. 4A). According to the instructions, CCRB should be removed after 12 h of placement regardless of whether the uterine balloon is discharged or not. In this study, the uterine balloons were discharged spontaneously only 25.6% cases in the control group and 20.4% cases in the study group at 12 h placement of CCRB. This means cervical dilation does not reach 2–3 cm in 75–80% of cases if CCRB is removed at this time. That is, the Bishop score improves by only 1 point or less in the dilation item and the total Bishop scores might still be less than 8 points; in such a condition the success rate of IOL with intravenous oxytocin is very low, which might lead to the anxiety of the pregnancies. In the present study, 6.84% of the patients requested cesarean section due to psychological factors in the control group, which was much higher than the rate of 0.93% in the study group.

We modified the operating process of CCRB in the following respects. First, we increased the volume of the uterine balloon to 120 mL, so that the maximum diameter of the balloon can reach 6 cm under the fetal head compression (Fig. 4B). Second, light traction was given to the catheter until it was discharged, and the added mechanical force increased the dilatation effect of the uterine balloon on the cervical canal, if the uterine balloon was not spontaneously discharged at 12 h of placement and there were no signs of infection. In this situation, even if the cervical dilatation retracted slightly after the uterine balloon was discharged, the dilation score can still be increased by 2–3 points and the Bishop score can reach more than 8 points, thereby improving the success rate of oxytocin induction. A significantly shorter length of first stage and full labor, and a distinct higher delivery rate within 24 h were observed in the study group. This could be attributed to higher cervix dilation (4–5 cm) by the time CCRB was discharged. Once uterine contractions were onset, labor quickly entered the active phase. The duration of labor was so short that many cases did not even require labor analgesia. This may also explain the significantly lower labor analgesia rate in the study group.

Other scholars have also noticed that the volume of the balloon might influence the induction to delivery time. Schoen CN et al. reviewed seven randomized controlled trials and concluded that women with a larger balloon volume had a significantly shorter time from induction to delivery [19]. In this study, we filled the uterine balloon to 120 mL and assisted the CCRB with light traction until the uterine balloon was discharged to facilitate a full dilation to the cervix. We did not use a larger volume considering the limited tension of the balloon and the potential risk of placental abruption. In addition, while increased uterine balloon volume could lift the fetal presentation, it does not benefit the engagement of the fetal head, and may increase the risk of umbilical cord prolapse.

Another modification of the operating process of CCRB was to reduce the volume of the vaginal balloon from 80 to 40 mL. The role of the vaginal balloon of CCRB was to fix the uterine balloon at the internal cervical os to achieve a full compression effect. A volume of 40 mL doesn’t reduce the diameter of the balloon too much, but might relieve vaginal foreign body sensation and avoid the occurrence of dysuria. Therefore, it increases the compliance of the patients.

The manufacturer’s instructions of CCRB recommend setting the catheter for no more than 12 h to prevent infection. With our modified procedure all the cases discharged the uterine balloon within 24 h. No maternal or fetal infection was observed in both groups. Peng J et al.’s research also showed that prolonging the balloon placement time to 24 h improved the cervical maturity without increasing the chance of infection [20].

When to perform amniotomy during IOL is controversial [21]. In this study, it was observed in the control group that rupture of membranes before labor could significantly shorten the duration of the first stage. In the study group, amniotomy before labor significantly shortened the induction to delivery time but did not distinctively change the duration of the first and total stages. We thought it might be due to the short stage of labor in the study group, so the effects of the timing of amniotomy were not obvious.

In this study, our modified operation of CCRB helped the cervix to dilate well, and effectively improved the Bishop score to 8 points or more. And following amniotomy and oxytocin induction, most patients entered the active phase quickly. The new protocol significantly improved the success rate of IOL and shortened the labor process. Stratified analysis showed that our modified protocol was even more effective in patients with poor initial cervical maturity (initial Bishop scores ≤ 3). In addition, lower volume of the vaginal balloon mitigates the incidence of vaginal foreign body sensation and dysuria, resulting in improve patient compliance.

This study also has some limitations. It was conducted in a general tertiary hospital, which could lead to some selection bias. In addition, the sample size of the study was relatively small. The results should be verified in larger sample studies in the future.

## Conclusions

In summary, this study modified the operating procedure of CCRB for cervical ripening. The modified method significantly improved the clinical efficiency, reduced the sensation of vaginal pressure, improved the delivery rate within 24 h, and shortened the length of hospital stay. It can improve patient compliance and reduce the cesarean section rate caused by psychological factors. The modified method could be beneficial from the perspective of health economics, and has good clinical application value and is worthy of promotion.

## Data Availability

The final trial datasets generated and/or analysed during the current study will be available in the ResMan repository. http://www.medresman.org.cn. And all data generated or analyzed during the current study are also available from the corresponding author on reasonable request.

## Abbreviations

IOL: Induction of labor
CCRB: COOK Cervical Ripening Balloon
BMI: Body mass index
HDP: Hypertensive disorders of pregnancy
PGDM: Pregestational diabetes mellitus
GDM: Gestational diabetes mellitus
